# Advances in management and treatment of tubercular meningitis – a narrative review

**DOI:** 10.1097/MS9.0000000000003348

**Published:** 2025-05-12

**Authors:** Manal Arshad Malik, Aleena Kamran, Dua Ahsan, Aafia Amjad, Sara Moatter, Amna Noor, Asharib Sohaib, Maryam Shaukat, Waniyah Masood, Muhammad Hasanain, Mohammed Mahmmoud Fadelallah Eljack

**Affiliations:** a Liaquat National Hospital, Karachi, Pakistan; b Jinnah Medical and Dental College, Karachi, Pakistan; c Sir Syed College of Medical Sciences, Karachi, Pakistan; d Isra University, Hyderabad, Pakistan; e Sindh Medical College, Jinnah Sindh Medical University, Karachi, Pakistan; f Dow Medical College, Dow University of Health Sciences, Karachi, Pakistan; g Aga Khan University, Karachi, Pakistan; h Dow University of Health Sciences, Karachi, Pakistan; i Community Department, University of Bakht Alruda, Ad Duwaym, Sudan

**Keywords:** drug resistance, PCR assays, tubercular meningitis (TBM)

## Abstract

**Background:**

Tuberculosis (TB), caused by *Mycobacterium tuberculosis*, continues to be a major global health issue, particularly due to its potential to cause severe complications such as tubercular meningitis (TBM), which is a fatal condition that proves difficult to diagnose and treat effectively and often results in poor outcomes, especially in children, due to delayed diagnosis, drug resistance, and limited diagnostic techniques.

**Methods:**

This review provides a comprehensive overview of recent advancements in TBM management and treatment. A systematic search was performed across major databases, including PubMed, Google Scholar, ClinicalTrials.gov, the WHO International Clinical Trials Registry Platform, and the ISRCTN Registry. The search strategy used terms like (“tubercular meningitis” OR “TBM” OR “TB meningitis”) AND (“diagnosis” OR “treatment” OR “clinical trials”). Inclusion criteria focused on studies published from January 2014 to September 2024, highlighting novel diagnostics, therapeutic advances, and clinical trials for TBM. Exclusion criteria involved studies unrelated to TBM or older than 10 years.

**Results:**

Diagnostic methods for TBM, such as microbiological and molecular techniques (Fig. 1), vary in sensitivity, with polymerase chain reaction assays being the most sensitive. While anti-TB drugs are available, drug resistance and poor cerebrospinal fluid penetration limit effectiveness. New molecular diagnostics and therapies, including anti-TNF agents, anti-inflammatory drugs, and antibiotics, show promise for improving outcomes.

**Figure 1.**
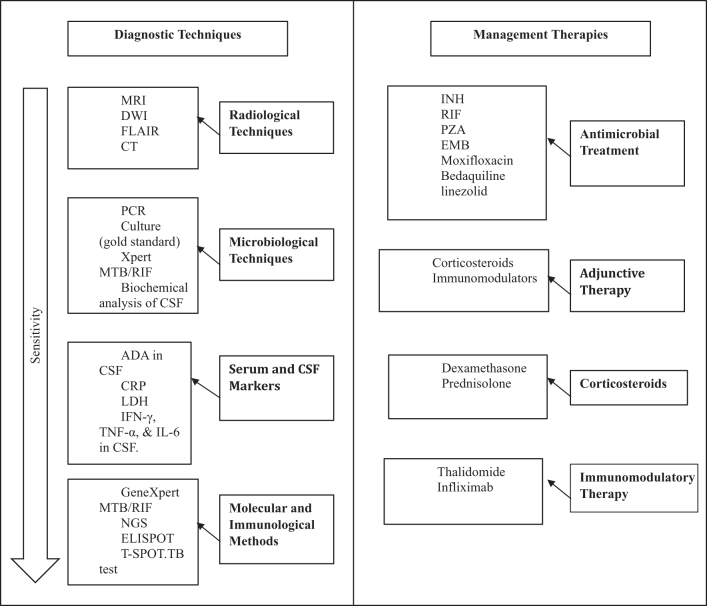
A summary of the diagnosis and management of tuberculosis meningitis.

**Conclusion:**

Despite recent advancements in TBM diagnostics and treatment, substantial challenges remain, particularly in addressing drug resistance and improving drug efficacy in the central nervous system. Continued innovation in molecular diagnostics and treatment approaches is essential to enhance TBM care and mitigate its devastating consequences.

## Introduction

Tuberculosis (TB) is a significant global health issue caused by *Mycobacterium tuberculosis*, a bacterium that has coexisted with humans since the prehistoric era^[^1^]^. It spreads through inhalation of airborne aerosol particles and primarily affects the lungs, leading to cough, fever, and chest pain^[^2^]^. However, TB can disseminate to other body parts, such as lymph nodes, genital, intestinal, and osteoarticular systems, through hematogenous and lymphatic routes^[^3^]^. According to the 2023 World Health Organization (WHO) report, 1.3 million people died from TB in 2022; moreover, TB is prevalent worldwide, affecting all countries and age groups The major risk factors of TB include young men, individuals living in developing or underdeveloped nations, healthcare workers, immunocompromised individuals due to HIV, and smokers^[^4^]^. The most deadly and damaging form of TB is tuberculosis meningitis (TBM)^[^5^]^. Failure of the immune system to eradicate TB can lead to hematogenous dissemination to distant sites, including the brain and meninges, where the pathogen may reproduce and cause local inflammation, termed as tuberculosis meningitis^[^6^]^. Signs and symptoms of TBM include altered mental state, seizures, cranial nerve palsies, and focal neurological deficits^[^7^]^. It causes mortality and disability and has a particularly high rate of poor outcomes in children. The main causes of these results are delays in diagnosis, treatment, and strong drug resistance^[^5^]^. Traditional microbiological and molecular techniques offer rapid results but are 10%–15% sensitive compared to culture tests that are 50%–60% sensitive but rather slow^[^7^]^. The most effective and rapid test, polymerase chain reaction (PCR)-based assay, is still 70% sensitive^[^7^]^. Second, no desirable procedure is established for treating drug-resistant TBM; furthermore, only a few published articles address customized drug-resistant TBM procedures and their effective concentration in the plasma and cerebrospinal fluid (CSF)^[^8^]^. These are the reasons why even though TBM is curable, it is usually not treated on time; therefore, it is crucial to improve and update current medical practices based on the latest research to improve the management and treatment of TBM, hence preventing unreasonable deaths. The objective of this research is to review the progress in the management and treatment of tuberculosis meningitis, which has been made by discussing the new diagnostic techniques, advances in treatment and antibiotics, and challenges that still need to be addressed.

**HIGHLIGHTS**

- Polymerase chain reaction and next generation sequencing (NGS) enhance TBM diagnosis but struggle with drug-resistant cases.
- TBM treatment faces drug resistance; high-dose rifampin and immunomodulators are vital.
- Trials like INSHORT and INTENSE-TBM offer hope in reducing TBM mortality and complications.
- TBM care demands multidisciplinary therapy, neurological monitoring, and rehabilitation.

## Pathophysiology

Tuberculous meningitis is an extended form of TB involving the meninges, which are protective brain and spinal cord coverings. TB first starts in the lungs, which are the primary site of infection^[^9^]^. Mycobacterium lodges in the lungs and alveoli when inhaled. Sometimes it is asymptomatic, and sometimes it is symptomatic. In the lungs, it forms a Ghon complex, a lung parenchymal lesion that may also involve lymph nodes. These bacteria have the ability to survive within the lungs and replicate within the lung macrophages because of their ability to evade host immune response. From its primary site, bacteria enter the blood to invade other body parts.

The immune system of our body responds to mycobacterium entry into the body by forming granulomas, which are also called tubercles. Granulomas are infectious aggregates of macrophages, lymphocytes, and the body’s immune cells^[^10^]^.

### Hematogenous spread

It is crucial for the development of extrapulmonary TB. During active TB of the lungs or other forms of disseminated TB, bacteria enter the bloodstream. Hematogenous spread is mostly triggered in patients with low immunity^[^11^]^.

### Central nervous system involvement

Bacteria then from the blood enter the central nervous system (CNS) and infect the meninges, which is its site of inflammation and infection. Bacteria adhere to the endothelial cells and then migrate across the blood-brain barrier. Bacteria traverse the barrier directly through infected endothelial cells or likely by transcytosis. This leads to several neurological changes and symptoms of tuberculous meningitis.

### Invasion of meninges

After crossing the blood-brain barrier, bacteria infect the meninges. The infection starts in the subarachnoid space and can spread to the pia and dura mater. Inflammatory response starts, and activation of macrophages occurs. T-cells and pro-inflammatory cytokines are released in response to bacterial invasion. Inflammatory response leads to the formation of granulomas in the meninges of the brain^[^12^]^.

### Granuloma formation

Granulomas are localized aggregates and are also called tuberculomas. These are formed within the brain parenchyma and the meninges^[^11^]^.

### Neurovascular compromise

Vascular inflammation may lead to ischemia in affected areas of the brain, causing focal neurological deficits, stroke, seizures, or altered consciousness, depending on the brain regions involved.

When mycobacterium invades meninges, CSF changes occur. There are elevated protein levels, reduced glucose levels and increased white blood cells in which lymphocytes are predominant. The patient presents with symptoms like headache, fever, neck stiffness, altered mental status, and possibly focal neurological signs. Hydrocephalus and cerebral infarcts may develop as complications. Early diagnosis and prompt treatment are required to avoid complications and manage the severe form of TB.

## Clinical manifestations

### Initial presentation and progression of TBM

**Figure 2.**
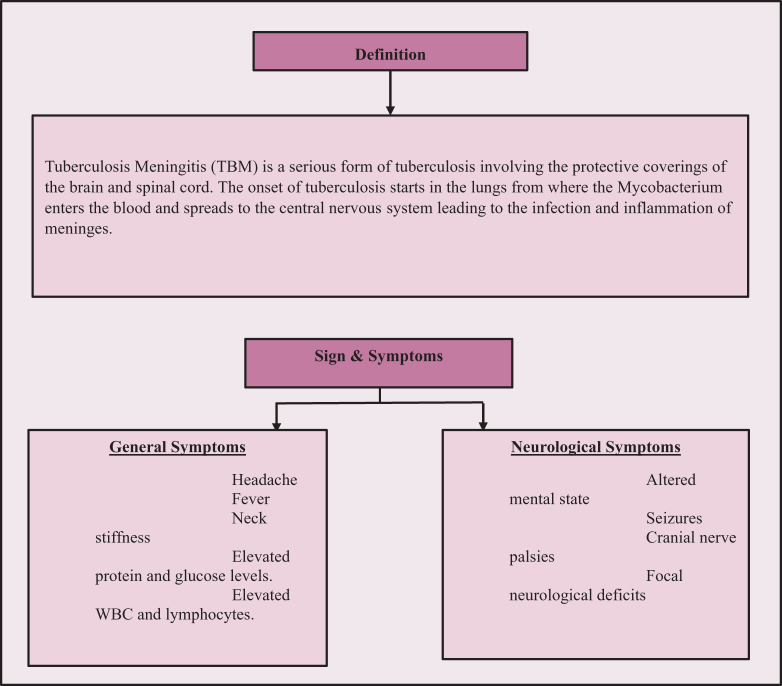
Signs and symptoms of tuberculosis meningitis.

Tuberculosis meningitis initially presents as a subacute illness with mild viral-like symptoms that gradually progresses over time^[^13^]^. These include fever, general discomfort, loss of appetite, muscle pain, and headaches (Fig. 2) that persist for days and weeks before more specific symptoms appear^[^14^]^. Less frequent early symptoms include constipation, as reported in 10% of the cases and diarrhea occurring a few times^[^15^]^. In contrast to bacterial meningitis, TBM symptoms manifest more slowly. As the disease progresses, headaches worsen, along with the appearance of nausea, vomiting, neck stiffness, and altered sensorium. The majority of the patients complain of continuous low-grade pyrexia and night sweats^[^14^]^. Headaches and mental status changes are more common in the elderly^[^16^]^.

With the advancement of TBM, meningeal irritation becomes evident, leading to seizures and signs of raised intracranial pressure (ICP). Children may present with bulging anterior fontanelles and sunsetting signs. Computed tomography (CT) imaging often reveals hydrocephalus in about 70% of the cases, usually a communicating one. This occurs when basal exudate causes an obstruction of the CSF pathways at the level of the tentorium. In rarer cases, basal exudates might obstruct the outflow of the fourth ventricle, leading to a noncommunicating type^[^13^]^. Cranial nerve palsies are common and mainly involve oculomotor issues. Other palsies include optic, abducens, and auditory nerve^[^17^]^. Some patients may suffer vision loss due to bilateral damage to optic nerves. This can happen as a consequence of optochiasmatic arachnoiditis, compression of optic chiasma secondary to hydrocephalus, optic nerve granuloma, or ethambutol toxicity. A physician may detect papilloedema during an ophthalmoscopic examination^[^14^]^.

### Neurological manifestations and complications

Neurological manifestations are prominent in the advanced stages, characterized by movement disorders resulting from basal ganglia infarction, such as choreiform or hemiballistic movements, athetosis, tremors, and myoclonic jerks. Children are more susceptible to tremors and abnormal movement than adults^[^16^]^. TBM could spread to the spine and cause various limb-related focal neurological defects, including monoplegia, hemiplegia, and paraplegia. Hyponatremia could occur due to the syndrome of inappropriate antidiuretic hormone or “cerebral salt wasting syndrome”^[^18^]^. Strokes involving anterior and middle cerebral arteries are a common and severe complication associated with the local inflammatory exudates^[^13^]^. If untreated, lethargy and confusion can progress to coma, especially in the elderly. Patients with HIV are prone to have extra meningeal involvement, such as respiratory, genitourinary, and abdominal^[^19^]^. In some cases, CNS tuberculomas may develop in association with TBM. They also manifest with focal neurologic signs or raised ICP due to obstruction of CSF pathways. Intracranial tuberculous abscesses present in a similar way and are more common in immunocompromised individuals^[^20^]^.

## Diagnostic techniques

Various diagnostics have led to advances in the treatment and management of tuberculous meningitis. This technology plays an important role in the timely and accurate diagnosis of tuberculous meningitis, which is important for initiating appropriate treatment and improving patient outcomes with the main diagnostic criteria defined in the literature as mentioned below.

### Radiological techniques

Magnetic resonance imaging (MRI) is the preferred test for the diagnosis of tuberculous meningitis (TBM) because it demonstrates important features that are often associated with TBM, such as basal meningeal cells, tuberculoma, hydrocephalus, and infarction. The introduction of gadolinium-enhanced MRI has improved the visualization of meningeal inflammation and brain tuberculomas. In addition, advanced MRI techniques such as diffusion-weighted imaging and fluid-attenuated inversion recovery sequences provide improved diagnostic rates and help distinguish TBM from other types of meningitis^[^21^]^. Despite the advantages of MRI, CT scans are still useful tools, especially when MRI is unavailable. CT scans are important in identifying problems such as hydrocephalus, infarction, and calcification. The advent of high-resolution CT and image reconstruction software has improved the ability to detect early parenchymal changes and basilar enhancement, but CT still lacks the sensitivity of MRI in some cases. In particular, multislice computed tomography (MSCT) has shown promise in detecting TB tumors, especially in children. MSCT scans can reveal early symptoms, such as mild pain, which helps to rapidly diagnose the disease and allows for timely treatment to prevent serious complications^[^22^]^.

### Microbiological techniques

Microbiological methods play an important role in the diagnosis of tuberculous meningitis (TBM). PCR is a molecular technique used to detect *M. tuberculosis* DNA in CSF. This approach is especially important when traditional diagnostic methods such as smear microscopy fail because the number of TBM lesions is too small to be reliable. Recent developments, including real-time PCR and nested PCR, have increased the sensitivity and specificity of TBM testing. This PCR technology allows rapid detection of bacteria, usually within a few hours, compared to the weeks required by culture methods. Although time-consuming, culture procedures remain the gold standard for diagnosing TBM. Culture is important not only to confirm the presence of *M. tuberculosis* but also to test for resistance, which is essential for effective treatment planning. Advances in automated culture systems, such as the BACTEC MGIT 960, have simplified this process, reducing the time required for bacterial growth testing from weeks to days. These electronic systems also provide good culture results, especially when bacterial counts are low^[^23^]^. Additionally, CSF-based studies have incorporated new technologies such as cassette-based nucleic acid amplification testing (Xpert MTB/RIF). Testing showed that most patients were very positive for *M. tuberculosis*. Biochemical and cellular analysis of CSF remains an important tool in distinguishing different types of meningitis, including purulent, tuberculous, and viral meningitis, and further aids in the diagnosis and treatment of TBM.

### Serum and CSF markers

In the field of tuberculous meningitis (TBM) diagnostic markers, both blood and CSF markers play an important role. Adenosine deaminase (ADA) is an enzyme found in high levels in the CSF of patients with TBM and is helpful in diagnosis, especially in limited areas. Recent developments have expanded the use of ADA by combining it with other markers, such as C-reactive protein (CRP) and lactate dehydrogenase. This combination is now used in multiplex testing to increase diagnostic accuracy and provide more diagnostic images^[^23^]^. Increased levels of cytokines (such as interferon-gamma [IFN-γ], tumor necrosis factor-alpha [TNF-α], and IL-6) in the CSF are proximal to TBM and may be helpful in its diagnosis. Recent studies have focused on the use of cytokine panels to differentiate TBM from other viruses and influenza strains. The use of multiplexed cytokine assays is promising in this regard, as these tests allow simultaneous measurement of many cytokines, improving diagnostic accuracy. In addition, serum markers such as ADA and CRP in CSF have been investigated for their ability to aid in the diagnosis of tuberculous meningitis, particularly in children.

### Molecular and immunological methods

Molecular and immunological methods have advanced diagnosing and treating tuberculous meningitis (TBM). The GeneXpert MTB/RIF test is an important test that detects *M. tuberculosis* and rifampin resistance directly from CSF and provides results in just 2 hours. Newer versions of GeneXpert Ultra are more sensitive, especially in paucibacillary cases where infection rates are low, making it important for early diagnosis of TBM^[^5^]^. Another powerful molecular sequencing technology, this tool helps detect *M. tuberculosis* DNA and identify drug-resistance mutations directly from clinical samples. NGS identifies new mutations associated with antibodies, enabling more personalized treatments. It also provides insight into the genetic diversity of TB, impacting treatment. This ELISPOT test measures T-cell responses to TB-specific antigens and helps distinguish active from latent infection in TB. Recent developments in the T-SPOT.TB test has increased its sensitivity and specificity, making it particularly useful in diagnostic testing, which is not always adequate in immunocompromised patients^[^21^]^.

### Diagnostic challenges and advances

Diagnosis of tuberculous meningitis is problematic, especially in limited areas. A comprehensive evaluation, including detailed history, physical examination, and CSF examination, is essential for accurate and timely diagnosis. In addition, advances in diagnostic technology, such as nucleic acid amplification tests and new imaging modalities, have improved the rapid and accurate diagnosis of tuberculous meningitis^[^22^]^.

Tuberculous meningitis (TBM), a lethal and devastating complication of extrapulmonary TB, requires an integrated approach to treatment due to its fatality and potential for severe neurological outcomes. The complex nature of TBM requires a delicate balance between effective antimicrobial regimens and adjunctive therapies.

## Management

### Antimicrobial treatment

Antimicrobic therapy remains the cornerstone in managing tuberculous meningitis (TBM). The standard regimen being isoniazid (INH), rifampicin (RIF), pyrazinamide (PZA), and ethambutol (EMB) administered for 2 months, followed by 10 months of administering INH and RIF as continuation phase^[^24^]^. Among these, INH and RIF are considered the most important first-line agents due to their excellent CSF penetration and potent bactericidal activity. The high mortality rate due to RIF-resistant TBM strains highlights the importance of these agents^[^25^]^.

Studies conducted by Heemskerk *et al* and Vinnard *et al* reported monoresistance to INH and RIF and multidrug resistance (MDR) in Vietnam and New York, respectively, and suggest that high doses of levofloxacin along with RIF can improve the survival rates in MDR-TBM patients^[^26,27^]^.

Cresswell *et al*, in his study, highlights the inclusion of moxifloxacin as an alternative to EMB due to its better CSF penetration and bactericidal nature^[^28^]^. Additionally, bedaquiline and linezolid, originally designed for MDR-TB and despite having adverse effects, have shown promising results in managing TBM by penetration into the CNS^[^29^]^.

### Adjunctive therapy

TBM, because of its severity being challenging to treat, demands comprehensive treatment approaches beyond the standard antimicrobic therapy. Adjunctive therapy, particularly corticosteroids and immunomodulators, plays a crucial role in terms of clinical outcomes. Studies indicate that corticosteroids have constantly shown a reduction in inflammation, mortality, and neurological complications^[^30^]^. Immunomodulators such as thalidomide (a TNF-α inhibitor) and aspirin also enhance outcomes by modulating immune response and reducing persistent inflammation^[^31^]^. Statins and antioxidants have also shown promising results in reducing neural injury and enhancing recovery in TBM patients^[^32^]^. Hence, these studies emphasize the need for a multidisciplinary approach to managing TBM for a better outcome.

#### Corticosteroids

Corticosteroids have been widely studied due to their important role in managing TBM by modulating the inflammatory response and alleviating both mortality and complications of the disease. Evidence from randomized controlled trials (RCTs) has demonstrated that adding corticosteroids to anti-tuberculosis treatment (ATT) lowers mortality rates, decreases the risk of death or severe disability, and reduces disease relapse. The standard approach involves administering dexamethasone or prednisolone during the intensive phase of ATT. The WHO recommends initiating and gradually tapering corticosteroids over 6–8 weeks.^[^33^]^ Studies have shown that corticosteroids reduce short-term mortality in TBM by nearly one-quarter, particularly in HIV-negative patients^[^30^]^. On the other hand, a randomized controlled study by Donovan *et al*^[^30^]^ found no significant survival benefit from adjunctive dexamethasone in HIV-positive adults with TBM. In conclusion, combining dexamethasone with conventional ATT enhances the effectiveness of TBM treatment by minimizing its adverse reactions and improving its overall efficacy, so it helps to normalize CSF parameters, leading to better results^[^31^]^.

#### Immunomodulatory therapy

The excessive host-inflammatory response is a key contributor to disease progression and pathophysiology of TBM, mainly led by TNF-α and IFN-γ. Adjunctive corticosteroids, though, improve overall survival but do not prevent morbidity, leading toward a need to add immunomodulatory therapy and standard ATT to improve clinical outcomes.

Among TB patients, with or without HIV co-infection, thalidomide was found to be effective in decreasing TNF-α levels, which in turn alleviates wasting, inflammatory responses, and viral load in HIV-infected patients^[^32^]^. A study by Marais *et al*^[^34^]^ describes that despite adequate ATT and high-dose corticosteroids, all cases of TBM still experienced clinical deterioration until infliximab therapy was initiated, leading to rapid improvement with no reported adverse effects.

In conclusion, the management of TBM requires a multifaceted approach that includes a combination of anti-microbial with strategic adjunctive treatment. Future research and clinical trials will continue to refine these strategies and improve patient outcomes.

## Treatment therapies under trial

The management of tuberculous meningitis (TBM) has seen various small improvements, yet the mortality rates continue to be high^[^35^]^. Treatment of CNS Tuberculous mirrors that of pulmonary TB, comprising an initial intensive phase followed by a continuation phase, the critical difference being that the ideal drug regimen and duration of treatment phases for CNS TB are not clearly defined^[^36^]^ and are often restricted by the low CNS penetration of most drugs, which limits their effectiveness^[^4^]^. Additionally, initial adjuvant corticosteroid therapy with dexamethasone or prednisolone is recommended, tapered over a period of 6–8 weeks^[^33^]^. Ongoing trials in clinical settings are essential for investigating new treatment methods designed to enhance results and reduce the impact of this serious illness.

A search of ClinicalTrials.gov, ISRCTN, and the ICTRP registries identified 33 clinical trials in TBM over the last decade, some of which have been completed while others are still ongoing. The trials mostly concentrate on optimizing anti-TB treatments, which highlights the uncertainty surrounding the best drug regimen and duration^[^37^]^ along with the fact that the drug regimens currently recommended by the WHO have insufficient drug exposure in the CNS^[^35^]^ with newer treatments including other antibiotics, immunomodulatory and anti-inflammatory drugs still under investigation.

The INSHORT trial (NCT05917340) is evaluating a more intense short-term treatment plan for tuberculous meningitis (TBM) in adults. This plan includes high-dose rifampicin, moxifloxacin, linezolid, and corticosteroids. Sponsored by the Indian Council of Medical Research, the study specifically examines mortality and disability rates intending to improve outcomes for TBM patients whereas the INTENSE-TBM (NCT04145258) compares the standard treatment with high-dose rifampin and linezolid with or without aspirin. Both Aspirin and Linezolid are used as adjuncts for the management of TBM. Aspirin, while it doesn’t reduce mortality, has been shown to reduce the risk of stroke^[^38^]^ and brain infarcts^[^39^]^, as seen in a Vietnamese trial (NCT02237365). Linezolid, on the other hand, has a high CNS penetration^[^36^]^ and is used as an adjunct for severe, refractory, or drug-resistant TBM cases; however, a recent phase 2 RCT failed to show any improvement in the efficacy of adjunctive linezolid to rifampin-containing first-line TB meningitis regimens in rabbits and mice. The findings are consistent with the LASER-TBM clinical trial, which showed no reduction in mortality when linezolid was combined with high-dose rifampin. Additionally, the LASER-TBM trial revealed a higher cumulative rate of adverse outcomes, including death and adverse events, when linezolid and aspirin were used together^[^40^]^. Other anti-inflammatory agents, like indomethacin and ibuprofen, are also currently being tested.

Paradoxical reaction refers to the worsening of existing TB lesions or the development of new lesions following an initial clinical improvement while on appropriate antitubercular therapy^[^41^]^ and can result in significant neurological impairment and lead to death^[^42^]^ therefore, it needs to be dealt with appropriately. Corticosteroids are used to reduce brain inflammation but may impede the penetration of antituberculous antibiotics into the subarachnoid space. Consequently, an alternative supplementary anti-inflammatory treatment for TBM is clearly required^[^43^]^.

A study done on rabbits with mycobacterial meningitis found a correlation between the levels of TNF-α in the CSF and the progression of meningitis. The increase in TNF-α levels in each individual corresponded to the worsening of the infection and often occurred before the animals passed away^[^43^]^. Consequently, several trials like (NCT05590455) have assessed anti-TNF agents like thalidomide and infliximab to counteract the harmful inflammatory responses in TBM, with Adalimumab being under investigation as an alternative. Other immunomodulatory agents under investigation include Cyclophosphamide and Azathioprine.

While most drug therapies center on the adult population, trials like the SURE trial (CTRI/2020/02/023317) and (CTRI/2024/07/070282) investigate fluoroquinolones in hopes of improving outcomes in children. These trials reflect ongoing efforts to enhance the understanding and treatment of TB through innovative approaches and rigorous clinical evaluations. The outcomes of these studies are expected to contribute significantly to improving TB management and patient outcomes globally.

## Long-term management, rehabilitation, and supportive care

Tuberculous meningitis is itself a life-threatening condition when left untreated; its long-term management is also complex and requires a multidisciplinary approach, which may involve infectious disease specialists, neurologists, and rehabilitation teams. It is crucial for ensuring the best possible outcomes and minimizing long-term complications.

The long-term management of tuberculous meningitis (TBM) involves an extensive approach to manage the disease effectively, prevent its complications, and ensure the patients recover from it completely. The management includes:

### Drug therapy

The current WHO guidelines for treating TBM recommend a 2-month regimen of four drugs in the initial phase: isoniazid, rifampin, pyrazinamide, and ethambutol. The aim of this phase is to rapidly reduce the bacterial load. This is followed by a continuation phase, which usually extends for 7–10 months or can be even longer, depending on the patient’s response to the treatment and the severity of the disease. The continuation phase generally involves isoniazid and rifampicin^[^44^]^. In the case of drug-resistant TBM, based on drug susceptibility, a modified regimen is followed, which may include second-line drugs like fluoroquinolones, aminoglycosides, and others^[^45^]^. After a clinical trial on follow-ups at 3-18 months, it was noticed that steroids reduce death by almost one quarter hence, are used adjunctively with ATT to reduce the inflammation and additionally to prevent complications^[^29^]^. Prednisolone or dexamethasone is commonly used for an initial 6–8 months with gradual tapering.

### Monitoring and follow-up

Regular clinical monitoring of the neurological status of the patient, the response to treatment, and keeping a check on any side effects or complications is necessary. Hence, it is advised that a follow-up must be done after a certain duration. To monitor the resolution of tuberculomas, hydrocephalus, and other intracranial pathologies, periodic CT or MRI studies can be done^[^44^]^. To monitor the response to therapy, repeated CSF analysis can be done, especially if clinical deterioration is suspected.

### Rehabilitation and supportive care

The defects that develop because of TBM can further be managed by neurorehabilitation. Physical therapy helps manage motor deficits and improve strength, coordination, and mobility. For patients with speech and cognitive impairment, speech and cognitive therapy can help regain communication skills, memory, and other cognitive functions. For patients who develop seizures as a result of TBM, antiepileptic drugs should be advised. For overall recovery from disease and effective immune function, adequate nutrition is necessary, especially in patients with poor appetite and malnutrition^[^46^]^. This should be included as part of the long-term care plan for the patient. Along with that, for patients with comorbidities like HIV or diabetes, an integrated care plan should be considered for optimal control of these conditions, which is necessary to improve TBM outcomes^[^30^]^.

Due to a lengthy treatment regimen, it is necessary to ensure the patient’s adherence to it to prevent the relapse of the disease and development of drug resistance. This can be supported by patient education and counseling, regular follow-up visits, and use of directly observed therapy (DOT) when necessary. On the basis of a retrospective study, DOTS is found to be 90.9% effective^[^47,48^]^.

## Conclusion

In conclusion, TBM remains a formidable global health challenge, characterized by its severe complications and high mortality rates, particularly in populations such as children and immunocompromised individuals. Despite advancements in diagnostic techniques and treatment strategies, significant gaps persist in managing this life-threatening condition. Early and accurate diagnosis is crucial, yet existing methods often fall short, highlighting the need for continued innovation in diagnostic technologies. The integration of advanced molecular techniques, such as PCR and NGS, alongside improved imaging modalities like MRI, holds promise for more timely and precise detection.

Therapeutic management of TBM continues to evolve, with ongoing research focusing on optimizing treatment regimens and exploring novel therapeutic options. The standard antimicrobic therapy, while foundational, is challenged by issues of drug resistance and inadequate CNS penetration. Recent trials investigating high-dose rifampicin, moxifloxacin, linezolid, and other adjunctive therapies offer the potential for improved outcomes, though results have been mixed. Corticosteroids and immunomodulators have shown promise in reducing inflammation and enhancing recovery, but their role remains complex and requires further validation.

Long-term management of TBM necessitates a multidisciplinary approach, integrating antitubercular therapy, supportive care, and rehabilitation to address both the immediate and residual effects of the disease. Effective management relies on adherence to treatment, monitoring for complications, and providing comprehensive care to improve patient outcomes. As research continues and new treatments are explored, it is imperative to adapt and refine strategies to mitigate the impact of TBM, ultimately aiming to reduce morbidity and mortality and improve the quality of life for affected individuals.

### Limitations

This narrative review has several limitations. It primarily includes studies published in English, which may have led to the exclusion of valuable research in other languages. Additionally, the lack of a comprehensive search strategy may have resulted in the omission of relevant studies. Furthermore, the review focuses only on studies and trials from the past 10 years, potentially overlooking earlier but still significant research.

## Data Availability

Data sharing does not apply to this article as no new data were created or analyzed in this study.

## Clinical trials information

Trial registry: ClinicalTrials.gov, ISRCTN, ICTRP; Trials reported: INSHORT trial (NCT05917340), INTENSE-TBM (NCT04145258), Vietnamese trial (NCT02237365), Trial (NCT05590455), SURE trial (CTRI/2020/02/023317), SURE trial (CTRI/2024/07/070282).
